# Decreased Bioimpedance Phase Angle in Patients with Diabetic Chronic Kidney Disease Stage 5

**DOI:** 10.3390/nu11122874

**Published:** 2019-11-25

**Authors:** Byoung-Geun Han, Jun Young Lee, Jae-Seok Kim, Jae-Won Yang

**Affiliations:** Department of Nephrology, Yonsei University Wonju College of Medicine, Wonju, Kang-won 26426, Korea; neptune@yonsei.ac.kr (B.-G.H.); junning@naver.com (J.Y.L.); ripplesong@hanmail.net (J.-S.K.)

**Keywords:** albumin, chronic kidney disease, diabetes type 2, impedance, inflammation, nutrition

## Abstract

Early detection and regular monitoring of the nutritional status of patients with diabetic chronic kidney disease (DMCKD) with reliable tools are necessary. We aimed to determine the clinical significance of the phase angle (PhA) in patients with DMCKD stage 5 not undergoing dialysis. A total of 219 patients (non-diabetic CKD stage 5 [nDMCKD5], *n* = 84; diabetic CKD stage 5 [DMCKD5], *n* = 135) were analyzed. The nDMCKD5 group had a significantly higher PhA (*p* = 0.001), intracellular water/body weight (*p* = 0.001), and albumin level (*p* = 0.010) than the DMCKD5 group. The DMCKD5 group experienced significantly more overhydration (*p* < 0.001). The PhA was positively associated with the lean tissue index (LTI) (*r* = 0.332; *p* < 0.001), hemoglobin level (*r* = 0.223; *p* = 0.010), albumin level (*r* = 0.524; *p* < 0.001), and estimated glomerular filtration rate (eGFR; *r* = 0.204; *p* = 0.018) in the DMCKD5 group. Multivariate logistic regression analysis showed the eGFR (odds ratio [OR]: 0.824, 95% confidence interval [CI]: 0.698–0.974); *p* = 0.023), LTI (OR: 0.771, 95% CI: 0.642–0.926; *p* = 0.005), and albumin level (OR: 0.131, 95% CI: 0.051–0.338; *p* < 0.001) were significantly associated with undernutrition (PhA < 4.17°) in the DMCKD5 group. Our observations suggest that the PhA could be used as a marker to reflect the nutritional status in patients with DMCKD5.

## 1. Introduction

Diabetes or chronic kidney disease (CKD) could be contributing to a poor prognosis related to nutritional status even though the two diseases are common, heterogeneous, and multifactorial. In fact, the prognosis for patients with diabetic chronic kidney disease (DMCKD) is considered to be worse than that for either disease individually. Reduced nutritional supply due to dietary restrictions, loss of appetite, nausea, and anorexia is one of the major contributors leading to undernutrition and waste of protein energy as uremia worsens in patients with DMCKD. However, from a nutritional point of view, there are no established recommendations as to when and how to diagnose nutritional deficiencies and how to initiate nutritional interventions for patients with DMCKD before renal replacement therapy.

Many nutritional assessment tools have been introduced. However, their use in CKD may be limited. The phase angle (PhA) is known as an indicator of cell integrity and a predictor of body cell mass, and has been suggested to be a prognostic, health, functional, and nutritional indicator of several diseases [1,2,3,4]. Of course, studies on the PhA have also been published in the area of kidney disease [5,6,7]. The PhA is a strictly objective rather than subjective measure. Previous PhA studies only included patients undergoing hemodialysis or peritoneal dialysis. Pre-dialysis CKD studies have also been performed on an extensive group of patients in all disease stages. However, in order to narrow down our focus group, we only analyzed the PhA in patients with CKD stage 5 (CKD5). In fact, reports that have evaluated and compared the nutritional status and body composition between non-diabetic CKD stage 5 (nDMCKD5) and diabetic CKD stage 5 (DMCKD5) patients at the same time are rare.

The main objectives of this research were to investigate the clinical differences in patients with diabetic and non-diabetic CKD5 not on renal replacement therapy and to evaluate the clinical significance of the PhA in patients with DMCKD5.

## 2. Materials and Methods

### 2.1. Patients and Data Collection

Since 2014, we have consecutively registered patients with CKD5 to a bioimpedance cohort. Therefore, the current study was a retrospective observational analysis of a prospective cohort database. Patients registered to the cohort underwent bioimpedance spectroscopy (BIS), echocardiography, and laboratory evaluation at the time of enrollment. Because dialysis treatment or kidney transplantation could affect the findings of the above-mentioned assessments, all patients were assessed just before the start of their first renal replacement therapy. Of the 227 total cohort patients, we excluded eight based on the following exclusion criteria: Renal cell carcinoma (*n* = 1), squamous cell carcinoma (lung, *n* = 1), hepatocellular carcinoma (*n* = 2), multiple myeloma (*n* = 1), type 1 diabetes (*n* = 1), and liver cirrhosis (*n* = 2).

In all patients, the BIS was performed using the BCM^TM^ (Body Composition Monitor^TM^, Fresenius Medical Care AG & Co., Bad Homburg vor der Höhe, Germany), which takes measurements at 50 different frequencies in the range of 5 to 1000 kHz. The PhAs were used as an indicator of the nutritional status. The PhA is an angle value of the time delay between the voltage waveform at 50 kHz and the current waveform. To determine the clinical significance of the PhA in patients with diabetes, the patients were classified into PhA ≥ 4.17° and PhA < 4.17° groups based on the median of the PhA in patients with DMCKD5. We evaluated the association of the PhA with standard objective nutritional indices such as the geriatric nutritional risk index (GNRI) and the prognostic nutritional index (PNI) rather than subjective indices. The GNRI is one of the most often used conventional nutritional indices [8]. Since the GNRI was developed to be suitable for the evaluation of the elderly, the PNI with relatively fewer age limitations was compared at the same time [9,10].

The patients were examined for structural and functional cardiac abnormalities such as coronary artery disease, atrial fibrillation, valvular heart disease, left ventricular diastolic dysfunction, and heart failure with a reduced ejection fraction. To assess the cardiac function and structure, the recommendations of the American Society of Echocardiography were used for the evaluation [11]. Echocardiography was performed by cardiologists who were completely blinded to the patient information using a 3-MHz transducer and commercial ultrasound system (GE Vivid-7; GE Healthcare, Chicago, IL, USA).

All laboratory studies were performed before the first application of dialysis. The high-sensitivity C-reactive protein (hs-CRP) level was measured using a Cobas 8000 modular analyzer (Roche Diagnostics GmbH, Mannheim, Germany). The normal range of the hs-CRP is below 0.3 g/dL (3 g/L). eGFR calculated using the MDRD formula was based on serum creatinine.

The study was carried out in accordance with the latest revision of the Declaration of Helsinki and approved by the Institutional Review Board of Yonsei University Wonju Severance Christian Hospital. We received written informed consent from all participant patients prior to entering the study.

### 2.2. Statistical Analysis

The study population characteristics are described as the mean ± standard deviation or percentage (%). The Student’s *t*-test and chi-squared test were used to determine the significance of differences in clinical variables between the groups of patients who were non-diabetic and diabetic. Pearson’s correlation coefficients were used to compare the PhA and the other potential explanatory variables between the groups of patients with and without diabetes as well as with the entire study population.

Multivariate logistic regression models were used to determine independent variables associated with undernutrition (PhA < 4.17°) in patients with DMCKD5. These variables were chosen considering collinearity among the factors that showed a significant correlation with the PhA. Odds ratios (OR), 95% confidence intervals (CI), and *p*-values were reported. The concordance statistic (c-statistic) was utilized to test the predictive accuracy of the logistic regression model. Goodness of fit for the model was assessed using the Hosmer–Lemeshow test, whereby we considered a value of *p* < 0.05 to indicate that the model had a poor fit. The level of significance was defined as *p* < 0.05. All analyses were performed using the IBM SPSS Statistics software (version 23.0; IBM Corporation, Armonk, NY, USA).

## 3. Results

### 3.1. Patient Characteristics

The final number of patients included was 219 (including 135 patients with diabetes, 61.6%) and their mean age was 60.32 ± 13.49 years. There were no significant differences in age and sex distribution between the patients with and without diabetes (Table 1). In the patients with diabetes, cardiac comorbidity was more common than with patients who did not have diabetes (*p* = 0.007). In addition, more diuretics and lipid lowering agents were given to the patients with diabetes. The primary renal disease in patients without diabetes is summarized in Table 1.

The PhA, intracellular water/body weight (ICW/BW), and albumin levels were significantly lower in patients with diabetes than in those without diabetes. The body mass index (BMI) and the variables reflecting volume status (overhydration (OH), overhydration/extracellular water (OH/ECW)) were significantly higher in the diabetic group. However, the hemoglobin, total cholesterol, triglyceride, calcium, phosphorus, and intact parathyroid hormone levels were similar between the two groups. Among the nutritional risk indices, only the PNI showed a significant difference between the two groups (Table 2).

### 3.2. Univariate Correlation Analysis

The PhA was positively correlated with the body cell mass (BCM), ICW/BW, and lean tissue index (LTI) in all patient groups. The PhA was also positively correlated with serum protein and albumin levels in patients both with and without diabetes. Similarly, the PhA showed a strong positive correlation with GNRI and PNI in all patient groups. The hs-CRP and magnesium levels showed a significant negative correlation with the PhA only in the patients with diabetes patients. In particular, the BMI showed a positive correlation with the PhA only in the patients without diabetes but not in patients with diabetes (Table 3).

### 3.3. Differences According to the Median PhA in Patients with DMCKD5

Among the BIS parameters, BCM, ICW/BW, and LTI were significantly greater in the well-nourished group (PhA ≥ 4.17°). The undernourished group (PhA < 4.17°) presented with lower scores of nutritional risk indices in both the GNRI and the PNI and with lower levels of hemoglobin, albumin, and eGFR. In contrast, indices reflecting the fluid balance (OH, OH/ECW) were significantly higher. The hs-CRP was also significantly higher in this patient group (Table 4).

### 3.4. Multivariate Analysis Using Logistic Regression in Patients with DMCKD5

Although the hs-CRP was correlated with the PhA in an unadjusted model, there was no significant relationship between the variables in the multivariate analysis. Among the significantly different variables between the well-nourished and undernourished groups, the eGFR, albumin level, and LTI remained in the multivariate analysis with statistical significance. These variables were chosen considering collinearity among the factors. The Hosmer–Lemeshow tests showed that the goodness of fit was significant in the multivariate analysis (*p* = 0.612). The c-statistic was 0.850 (95% CI 0.782–0.918; *p* < 0.001) in the multivariate analysis (Table 5).

## 4. Discussion

In general, reduced protein intake, limited sodium and potassium intake, and reduced phosphorus intake in patients with CKD are emphasized. However, there is still controversy as to the relative advantages and disadvantages of limiting protein intake to prevent the progression of CKD without causing protein energy wasting [12]. Nutritional therapy, which can prevent the deterioration of kidney function in patients with DMCKD, and even better control of blood sugar, blood pressure, and lipid profile, are also very important [13].

Diabetologists, nephrologists, and nutritionists should be involved in treating patients with DMCKD who are getting worse, because the exact assessments of the nutritional status of those patients and the proper nutritional interventions for those patients are never easy and simple [14]. Most importantly, dietary advice should be tailored to the individual and the stage of DMCKD. In clinical practice, there is a need for a noninvasive, objective, fast, and reproducible method to assess the nutritional status of patients with diabetes and/or CKD for a team approach.

The PhA is the angle of the vector formed by the body’s reactance and resistance. The PhA is specifically correlated with muscle mass, muscle strength, and frailty scales. In many diseases, the PhA is known to be linked to nutritional status and to predict morbidity and mortality for certain diseases [1,2,3]. The PhA can also reflect the nutritional status of patients with end stage renal disease (ESRD) undergoing maintenance hemodialysis or not on dialysis [5,6,7]. The PhA has been used to study other diseases, but few studies have been conducted exclusively on patients with DMCKD stage 5. Generally, a low PhA may suggest deterioration of the cell membrane and cellular dysfunction. A low PhA is associated with frailty [15,16,17] as well as being associated with sarcopenia [18,19,20]. In this study, there was a significant association between the PhA and OH/ECW, but we excluded OH/ECW from the multivariate analysis. The PhA was significantly associated with LTI and BCM when statistical analysis was performed separately for the whole patient group and DMCKD patient group (Table 3). On the other hand, OH/ECW was not associated with LTI (*p* = 0.200) and BCM (*p* = 0.069) in patients with DMCKD. Therefore, we thought that the PhA could better reflect nutritional status clinically.

The loss of muscle mass was also significantly related to the glomerular filtration rate decline [18]. Skeletal muscle wasting is associated with mortality and major adverse cardiovascular events in patients with ESRD [21]. Furthermore, undernutrition is a key contributor to the development of sarcopenia and frailty in those with CKD. Decreased muscle mass, strength, and function are also associated with diabetes and lead to frailty and disability, eventually [22]. Therefore, early detection of undernutrition and an appropriate nutritional approach across the stages of DMCKD is not only essential to obtain optimal healthy lifestyle, but also to reduce the risk of frailty and cardiovascular events. In the diabetic group of our study, the LTI was associated with the PhA, suggesting that the PhA could be a marker to predict sarcopenia and frailty.

The BMI was higher in the diabetic group compared to the non-diabetic group, but the LTI and fat tissue index (FTI) were not different between the two groups, which may be due to other factors. Considering that more patients in the diabetic group were taking diuretics and BMI did not reflect the volume status of the patients, this may be related to fluid overload. In our study, fluid overload was observed in the diabetic group.

Patient risk stratification based on nutritional status can provide useful prognostic information and identify those at high risk. The GNRI is one of the most commonly used nutritional indices [8]. The PNI has been reported to be associated with cardiovascular mortality in patients with incident peritoneal dialysis [23]. In this study, we used Onodera’s modified PNI calculated using serum albumin levels and peripheral lymphocyte counts [9]. The lymphocyte count may reflect the degree of inflammation or immune index. The lymphocyte count is one of the principal biohumoral parameters of undernutrition. Therefore, the PNI could be a marker predicting the risk of undernutrition. Both the GNRI and PNI were highly associated with the PhA. However, since albumin levels are implied in the GNRI and PNI values, GNRI and PNI were eventually excluded from the multivariate analysis in this study. Our findings suggest that the PhA could not only be a single marker to assess nutritional status, but potentially a member of a new combined risk indicator in the future.

Serum albumin level alone could not be used as a pure marker of the nutritional status in patients on dialysis because low albumin levels are often associated with chronic systemic inflammation and other factors that are not nutrition-related. Analysis of serum albumin levels for predicting mortality risk in ESRD should always be combined with the measurement of hs-CRP levels [24]. In our study, albumin levels showed an independent association with the PhA, but hs-CRP levels did not (Table 5). Therefore, our results suggest that it may be necessary to use a combination of the PhA and hs-CRP levels when testing models that predict sarcopenia, frailty, and/or mortality. Chronic inflammatory conditions, which are common in patients with DMCKD, have been recognized as a major contributor to cardiovascular disease, protein energy wasting, osteoporosis, sarcopenia, and frailty [25,26,27].

This study includes several limitations. First, this was a single-center study that included a relatively small number of patients and the design was a retrospective observational study. Second, not all patients with DMCKD had a kidney biopsy performed to confirm their diagnosis. Third, there was no assessment of the duration of diabetes, potentially leading to bias. Fourth, this study cannot be generalized to the other stage CKD populations. Fifth, serial changes in the PhA in relation to the removal of uremic toxins by renal replacement therapy were not assessable over time. Lastly, even though all patients received renal nutritional recommendations such as low protein diet, restriction of sodium, potassium, phosphate, and water intake, the amount of protein intake was not controlled, and the dietary intake was not assessed at the time of enrollment. In fact, patients immediately prior to dialysis requirement may be very heterogeneous with respect to fluid balance, food consumption, potential anorexia, taste changes, etc. However, we attempted to analyze a patient population (CKD stage 5) that is relatively homogenous in terms of eGFR. There was no significant difference in eGFR between the diabetic and non-diabetic groups in our study. We also objectively assessed the nutritional status of patients using the PhA. To our knowledge, this is the first study to evaluate the clinical significance of the PhA in patients with DMCKD5.

## 5. Conclusions

A biomarker that is not affected by hydration status, stage of renal function, and blood glucose levels in patients with DMCKD is ideal and necessary. Our observations suggest that the PhA, although influenced by hydration status, could be a marker that reflects nutritional status in patients with DMCKD. However, larger prospective studies are needed to determine the significance of the PhA to populations in other stages of DMCKD and the responsiveness of the PhA to interventions aimed at improving nutritional status.

## Figures and Tables

**Table 1:** Demographic characteristics of the study patients.

Variables	Total	nDMCKD5	DMCKD5	*p*-Value
		(*N* = 84)	(*N* = 135)	
Age, years				
<65	134 (61.2%)	48 (57.1%)	86 (63.7%)	0.333
≥65	85 (38.8%)	36 (42.9%)	49 (36.3%)	
Sex				
Males	125 (57.1%)	46 (54.8%)	79 (58.5%)	0.585
Females	94 (42.9%)	38 (45.2%)	56 (41.5%)	
Cardiac abnormalities				
Yes	129 (58.9%)	40 (47.6%)	89 (65.9%)	0.007
No	90 (41.1%)	44 (52.4%)	46 (34.1%)	
Diuretics				
Yes	142 (64.8%)	46 (54.8%)	96 (71.1%)	0.014
No	77 (35.2%)	38 (45.2%)	39 (28.9%)	
Anticoagulants				
Yes	99 (45.2%)	36 (42.9%)	63 (46.7%)	0.582
No	120 (54.8%)	48 (57.1%)	72 (53.3%)	
Lipid lowering agents				
Yes	121 (55.3%)	39 (46.4%)	82 (60.7%)	0.038
No	98 (44.7%)	45 (53.6%)	53 (39.3%)	
Primary renal disease				
Hypertension	18		-	
CGN	22		-	
FSGS	4		-	
IgAN	11		-	
MGN	1		-	
Hereditary/congenital disease	6		-	
Other	9		-	
Unknown	13		-	

CGN, chronic glomerulonephritis; DMCKD5, diabetic chronic kidney disease stage 5; nDMCKD5, non-diabetic chronic kidney disease stage 5; FSGS, focal segmental glomerulosclerosis; IgAN, immunoglobulin A nephropathy; MGN, membranous glomerulonephritis.

**Table 2:** Parameters compared between the non-diabetic and diabetic groups.

Variables	Total	nDMCKD5	DMCKD5	*p*-Value
		(*N* = 84)	(*N* = 135)	
Age, years	60.32 ± 13.49	61.64 ± 15.81	59.50 ± 11.81	0.286
Phase angle, °	4.31 ± 1.22	4.66 ± 1.28	4.10 ± 1.13	0.001
SBP, mmHg	141.16 ± 19.88	137.11 ± 19.90	143.79 ± 19.38	0.015
DBP, mmHg	79.53 ± 11.08	79.33 ± 12.39	79.70 ± 10.18	0.820
BMI, kg/m^2^	24.76 ± 4.04	23.50 ± 3.83	25.55 ± 3.99	<0.001
BCM, kg	18.63 ± 6.56	18.87 ± 6.57	18.44 ± 6.56	0.673
ICW/BW, L/kg	0.26 ± 0.05	0.27 ± 0.06	0.25 ± 0.04	0.001
LTI, kg/m^2^	12.95 ± 3.16	13.11 ± 3.15	12.85 ± 3.17	0.548
FTI, kg/m^2^	11.15 ± 9.87	11.08 ± 14.87	11.19 ± 4.65	0.938
GNRI	92.22 ± 8.72	93.57 ± 8.89	91.38 ± 8.55	0.070
PNI	34.64 ± 5.69	35.91 ± 5.51	33.83 ± 5.67	0.009
Hemoglobin, g/dL	9.13 ± 1.26	9.12 ± 1.35	9.14 ± 1.20	0.879
Total Protein, g/dL	6.14 ± 0.76	6.17 ± 0.69	6.13 ± 0.80	0.661
Albumin, g/dL	3.47 ± 0.57	3.59 ± 0.55	3.39 ± 0.56	0.010
Total cholesterol, mg/dL	148.18 ± 47.01	149.88 ± 46.03	147.13 ± 47.75	0.674
Triglyceride, mg/dL	129.93 ± 74.28	134.35 ± 96.17	127.81 ± 56.88	0.593
Glucose, mg/dL	144.54 ± 68.93	123.96 ± 41.20	157.35 ± 79.04	<0.001
Ccalcium, mg/dL	7.67 ± 1.06	7.72 ± 1.15	7.64 ± 1.00	0.597
P, mg/dL	5.94 ± 1.60	5.99 ± 1.67	5.90 ± 1.56	0.677
Magnesium, mg/dL	2.32 ± 0.48	2.27 ± 0.40	2.35 ± 0.53	0.274
Uric acid, mg/dL	8.08 ± 2.40	7.75 ± 2.09	8.28 ± 2.57	0.121
iPTH, pg/mL	303.76 ± 206.06	331.71 ± 253.08	286.18 ± 168.73	0.150
hs-CRP, mg/dL	1.38 ± 2.87	1.27 ± 2.46	1.45 ± 3.11	0.656
eGFR, mL/min/1.73 m^2^	7.04 ± 2.71	6.75 ± 2.61	7.21 ± 2.77	0.225
OH, liter	2.86 ± 3.57	1.77 ± 2.66	3.54 ± 3.90	<0.001
OH/ECW, %	14.61 ± 14.63	9.99 ± 14.34	17.53 ± 14.12	<0.001

BCM, body cell mass; BMI, body mass index; BW, body weight; FTI, fat tissue index; DBPDBS, diastolic blood pressure; DMCKD, diabetic chronic kidney disease; nDMCKD, non-diabetic chronic kidney disease; eGFR, estimated glomerular filtration rate; GNRI, geriatric nutritional risk index; hs-CRP, high-sensitivity C-reactive protein; ICW, intracellular water; LTI, lean tissue index; PNI, prognostic nutritional index; iPTH, intact parathyroid hormone; SBPSBS, systolic blood pressure.

**Table 3:** Correlations between variables.

Variables	Phase Angle, All Patients		Phase Angle, Non-Diabetic		Phase Angle, Diabetic	
	Correlation Coefficient	*p*-Value	Correlation Coefficient	*p*-Value	Correlation Coefficient	*p*-Value
Age, years	−0.223	0.001	−0.512	<0.001	0.000	0.999
SBP, mmHg	−0.195	0.004	−0.234	0.032	−0.129	0.137
DBP, mmHg	−0.017	0.804	0.021	0.853	−0.069	0.427
BMI, kg/m^2^	0.074	0.274	0.291	0.007	0.039	0.655
BCM, kg	0.446	<0.001	0.639	<0.001	0.328	<0.001
ICW/BW, L/kg	0.440	<0.001	0.433	<0.001	0.391	<0.001
LTI, kg/m^2^	0.459	<0.001	0.659	<0.001	0.332	<0.001
FTI, kg/m^2^	0.049	0.470	0.074	0.506	0.021	0.810
GNRI	0.526	<0.001	0.492	<0.001	0.533	<0.001
PNI	0.518	<0.001	0.461	<0.001	0.524	<0.001
Hemoglobin, g/dL	0.168	0.013	0.115	0.299	0.223	0.010
Total Protein, g/dL	0.401	<0.001	0.303	0.005	0.472	<0.001
Albumin, g/dL	0.517	<0.001	0.461	<0.001	0.524	<0.001
Total cholesterol, mg/dL	−0.041	0.546	0.017	0.878	−0.096	0.272
Triglyceride, mg/dL	0.181	0.007	0.193	0.079	0.164	0.058
Glucose, mg/dL	0.033	0.627	0.016	0.887	0.127	0.142
Ccalcium, mg/dL	0.004	0.958	−0.111	0.316	0.081	0.354
P, mg/dL	−0.094	0.167	−0.092	0.405	−0.110	0.205
Magnesium, mg/dL	−0.137	0.049	−0.027	0.815	−0.182	0.039
Uric acid, mg/dL	−0.043	0.540	−0.200	0.076	0.082	0.358
iPTH, pg/mL	0.092	0.182	0.128	0.249	0.003	0.972
hs-CRP, mg/dL	−0.208	0.003	−0.042	0.712	−0.307	0.001
eGFR, mL/min/1.73 m^2^	0.137	0.043	0.095	0.390	0.204	0.018
HbA1C, %	-	-	-	-	0.088	0.359

BCM, body cell mass; BMI, body mass index; BW, body weight; FTI, fat tissue index; DBPS, diastolic blood pressure; DMCKD, diabetic chronic kidney disease; nDMCKD, non-diabetic chronic kidney disease; eGFR, estimated glomerular filtration rate; GNRI, geriatric nutritional risk index; HbA1C, hemoglobin A1C; hs-CRP, high-sensitivity C-reactive protein; ICW, intracellular water; LTI, lean tissue index; PNI, prognostic nutritional index; iPTH, intact parathyroid hormone; SBPS, systolic blood pressure.

**Table 4:** Compared parameters according to the median of the phase angle in patients with DMCKD5.

Variables	Phase Angle < 4.17°	Phase Angle ≥ 4.17°	*p*-Value
	(*N* = 66)	(*N* = 69)	
Age, years	59.49 ± 13.45	59.30 ± 10.09	0.928
Phase angle, °	3.09 ± 0.58	5.03 ± 0.59	<0.001
SBP, mmHg	147.38 ± 20.15	140.88 ± 18.55	0.053
DBP, mmHg	80.71 ± 11.47	79.17 ± 9.45	0.396
BMI, kg/m^2^	25.22 ± 4.21	25.83 ± 3.74	0.377
BCM, kg	16.34 ± 6.70	20.45 ± 5.79	<0.001
ICW/BW, L/kg	0.24 ± 0.04	0.27 ± 0.04	<0.001
LTI, kg/m^2^	11.81 ± 3.36	13.81 ± 2.65	<0.001
FTI, kg/m^2^	11.10 ± 4.69	11.29 ± 4.62	0.814
GNRI	87.44 ± 8.22	95.08 ± 7.11	<0.001
PNI	31.23 ± 5.28	36.16 ± 4.83	<0.001
Hemoglobin, g/dL	8.90 ± 1.06	9.37 ± 1.29	0.024
Total Protein, g/dL	5.80 ± 0.74	6.42 ± 0.74	<0.001
Albumin, g/dL	3.13 ± 0.52	3.62 ± 0.48	<0.001
Total cholesterol, mg/dL	146.42 ± 54.81	147.13 ± 40.44	0.931
Triglycerides, mg/dL	113.63 ± 49.63	139.70 ± 59.76	0.007
Glucose, mg/dL	148.72 ± 66.42	166.07 ± 89.43	0.199
Calcium, mg/dL	7.54 ± 0.86	7.73 ± 1.11	0.283
P, mg/dL	6.17 ± 1.55	5.66 ± 1.53	0.061
Magnesium, mg/dL	2.43 ± 0.55	2.26 ±0.49	0.065
Uric acid, mg/dL	8.27 ± 2.54	8.25 ± 2.60	0.967
iPTH, pg/mL	294.89 ± 174.00	282.37 ± 163.93	0.671
hs-CRP, mg/dL	2.11 ± 3.90	0.84 ± 1.94	0.027
eGFR, mL/min/1.73 m^2^	6.47 ± 2.62	7.84 ± 2.70	0.003
OH, liter	5.57 ± 4.46	1.59 ± 1.70	<0.001
OH/ECW, %	27.07 ± 12.30	8.46 ± 8.49	<0.001
HbA1C, %	6.78 ± 1.41	7.23 ± 1.88	0.157

BCM, body cell mass; BMI, body mass index; BW, body weight; FTI, fat tissue index; DBPS, diastolic blood pressure; DMCKD, diabetic chronic kidney disease; ECW, extracellular water; nDMCKD, non-diabetic chronic kidney disease; eGFR, estimated glomerular filtration rate; GNRI, geriatric nutritional risk index; hs-CRP, high-sensitivity C-reactive protein; ICW, intracellular water; LTI, lean tissue index; PNI, prognostic nutritional index; iPTH, intact parathyroid hormone; OH, overhydration; SBPS, systolic blood pressure.

**Table 5:** Factors independently associated with PhA ≥ 4.17° in patients with DMCKD5.

Variables	Univariate Analysis		Multivariate Analysis-Adjusted	
	OR (95% CI)	*p*-Value	OR (95% CI)	*p*-Value
Age, years	1.001 (0.973–1.031)	0.926	0.998 (0.959–1.038)	0.920
Sex, males	2.743 (1.355–5.554)	0.005	1.284 (0.458–3.602)	0.634
Diuretics use, yes	1.191 (0.564–2.515)	0.646	1.955 (0.652–5.862)	0.232
eGFR, mL/min/1.73 m^2^	0.810 (0.699–0.939)	0.005	0.824 (0.698–0.974)	0.023
Albumin, g/dL	0.138 (0.060–0.319)	<0.001	0.131 (0.051–0.338)	<0.001
LTI, kg/m^2^	0.792 (0.694–0.903)	<0.001	0.771 (0.642–0.926)	0.005
hs-CRP, mg/dL	0.182 (1.003–1.392)	0.046	1.096 (0.914–1.315)	0.323

CI, confidence interval; eGFR, estimated glomerular filtration rate; hs-CRP, high-sensitivity C-reactive protein; LTI, lean tissue index; OR, odds ratio.
